# T‐cell exhaustion from a multiomics perspective: Differentiation mechanisms and regulatory networks in the journey from progenitor‐Exhausted T cells to terminally exhausted T cells

**DOI:** 10.1002/ctm2.70609

**Published:** 2026-02-02

**Authors:** Tong Zhu, Xiaoyu Teng, Qinlian Jiao, Yidan Ren, Yunshan Wang, Maoxiao Feng

**Affiliations:** ^1^ Department of Clinical Laboratory Shandong Provincial Hospital Affiliated to Shandong First Medical University Jinan Shandong China; ^2^ Department of Clinical Laboratory Shandong Provincial Hospital Shandong University Jinan Shandong China

**Keywords:** T‐cell exhaustion, single‐cell multiomics, Tpex, terminally exhausted T cells, transcriptomics, epigenomics, metabolomics, proteomics, PTM proteomics, cancer immunotherapy

## Abstract

A central hurdle limiting the success of T‐cell‐based immunotherapies is the progressive dysfunction of T cells, known as exhaustion. Overcoming this exhausted state is therefore a pivotal objective in translational oncology and immunology. The advent of single‐cell multiomics has fundamentally revised the once‐prevailing view of exhaustion as a uniform endpoint. Instead, it is now recognised as a dynamic differentiation process comprising a spectrum of distinct cellular states. This spectrum is organised along a hierarchical axis, originating from progenitor‐exhausted (Tpex) cells that retain proliferative potential and advancing towards terminally exhausted (Tex) populations with severely impaired effector functions. We undertake a comprehensive synthesis of multiomics data—spanning transcriptomic, epigenomic, metabolomic, proteomic and posttranslational modification (PTM)‐proteomic layers—to decipher the interconnected regulatory programmes that dictate commitment along this exhaustion axis. From this integrated analysis, we derive a unified mechanistic framework that delineates the molecular drivers of Tpex cell fate determination and terminal exhaustion. Beyond its explanatory power for basic biology, this framework serves as a direct roadmap for therapeutic innovation, highlighting novel nodes for intervention aimed at reinvigorating the exhausted T‐cell compartment. The practical application of these insights holds significant promise for enhancing the efficacy of established current immunotherapeutic platforms.

**Key points:**

- This review is the first to integrate multi‐omics evidence for constructing a dynamic regulatory map of T‐cell exhaustion.
- It highlights the critical cross‐omics synergistic mechanisms, such as metabolic reprogramming influencing epigenetic remodeling to drive cell fate.
- The multi‐omics perspective presented directly informs novel therapeutic strategies.

## Graphical abstract

**(1) Core Process**: Persistent antigen, from TDLNs to TME, drives Tpex → Tex‐int → Tex‐term differentiation. **(2) Multiomics Regulation**: This fate transition is orchestrated by a co‐ordinated, multi‐layered molecular network. **(3) Functional Outcome**: Dysregulated signalling and surging co‐inhibitory receptors lock in proliferative arrest and functional loss. **(4) Therapeutic Strategies**: Rational combinations (ICB, epigenetic drugs, engineered T cells) target this network.

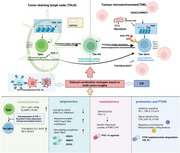

## 1 INTRODUCTION

T‐cell exhaustion, a state of progressive functional impairment in CD8^+^ T cells, is primarily mediated by chronic antigen exposure, as occurs in persistent infections and cancer, ultimately culminating in severe dysfunction.^1^, ^2^, ^3^ This exhausted phenotype is defined by a constellation of functional and molecular alterations. Key functional impairments include a progressive decline in cytotoxic effector functions, diminished self‐renewal capacity and metabolic dysregulation.^1^, ^2^, ^4^, ^5^ Persistent, high‐intensity antigenic stimulation is the primary driver of exhaustion, being its most prominent hallmark.^1^, ^6^ As T cells progress from a progenitor‐exhausted (Tpex) state towards complete functional impairment, they undergo extensive reorganisation of their transcriptomic and epigenomic landscapes.^7^, ^8^, ^9^

The exhausted state in CD8^+^T cells was long considered a relatively homogeneous population, representing a fixed endpoint of differentiation that was largely irreversible. However, this conventional view has been fundamentally revised due to the transformative insights provided by single‐cell sequencing and multi‐omics technologies.^10^, ^11^ Recent studies have delineated a complex architecture and significant diversity of cellular states within the exhausted T‐cell compartment, which comprises several discrete subpopulations. Foremost among them are two functionally distinct populations: Tpex and Tex cell subsets.^12^ Tpex cells are defined by a core transcriptional signature that includes factors such as TCF‐1. This molecular programme underpins their stem‐like properties: the ability to self‐renew and to differentiate into various effector lineages. The persistence of antitumour immune responses is mediated in part by the continued presence of this Tpex cell population.^13^, ^14^ The self‐renewal of Tpex cells ensures their population persists and continuously generates new effector T cells, which is indispensable for maintaining long‐term immune responses.^15^ In stark contrast, terminally exhausted (Tex) T cells are defined by a profound loss of effector capacity. This dysfunctional state, representing the final and most severe stage of T cell exhaustion, is characterised by two hallmark features: a profound loss of effector capacity coupled with the simultaneous upregulation of a broad repertoire of IRs on the cell surface.^1^, ^5^, ^16^

The existence of Tpex cells provides a crucial cellular basis for explaining the sustained clinical efficacy of immune checkpoint blockade (ICB)—the success of these treatments relies heavily on a pre‐existing reservoir of Tpex cells with regenerative capacity.^17^, ^18^, ^19^ Therefore, a deep understanding of the mechanisms sustaining Tpex cells and the regulatory pathways governing their differentiation into terminal exhaustion is crucial for unlocking this biology. This understanding directly informs the design of new therapeutic approaches aimed at improving current immunotherapies.^20^

This review aims to synthesise recent findings from transcriptomics, epigenomics, metabolomics, proteomics and post‐translational modification proteomics analyses to construct a comprehensive molecular atlas that maps the formation, differentiation and functional fate decisions of Tpex cells, culminating in their transition to a Tex state.

## 2 HETEROGENEITY AND BIOLOGICAL CHARACTERISTICS OF Tpex AND Tex T CELLS

Exhausted T cells display a defining set of attributes—including unique molecular signatures, altered effector functions, specialised metabolic profiles and distinct transcriptional and epigenetic regulatory programmes—that collectively establish them as a cell state distinct from effector T (Teff) cells.^2^

### 2.1 Molecular features of Tpex and Tex T cells

Tpex cells exhibit a core transcriptional signature dominated by TCF‐1 (*Tcf7*). This factor acts as a pivotal orchestrator, crucial for preserving stem‐like properties, preventing early terminal differentiation and establishing Tpex identity.^13^, ^14^, ^21^ Their molecular circuitry is further shaped by MYB, ID3, LEF1 and SLAMF6.^13^, ^14^ Key surface markers defining Tpex cells include positivity for TCF‐1, CXCR5, CD62L and SLAMF6, along with intermediate expression of the inhibitory checkpoint PD‐1 and minimal TIM‐3 and LAG‐3.^22^, ^23^

In contrast, the transcription factor TOX, expressed at high levels in Tex cells, serves as a primary driver of the exhaustion programme. It initiates and stabilises this state through extensive epigenetic reprogramming.^24^, ^25^ The transcriptional network in these cells additionally involves factors including the NR4A family (NR4A1–3), BATF and BLIMP‐1.^26^, ^27^ Their surface marker profile is defined by low or absent TCF‐1 expression, loss of CXCR5 expression and high expression of CD39 and CD101. Furthermore, a defining feature of these cells is the elevated co‐expression of PD‐1, TIM‐3, LAG‐3, TIGIT and CTLA‐4.^2^

### 2.2 Functional characteristics of Tpex and Tex T cells

The defining functional properties of this progenitor population encompass self‐renewal, proliferative potential and the capability to give rise to further differentiated states within the exhausted T‐cell lineage. They retain a limited capacity to produce effector cytokines and express intermediate levels of cytotoxic molecules, including granzyme and perforin.^28^ The Tex state constitutes the definitive and final stage in the T‐cell exhaustion differentiation hierarchy. They have lost their self‐renewal and proliferative capabilities and exhibit severe functional impairments. These cells are unable to produce key effector cytokines, exhibit diminished cytotoxicity and display high expression of co‐IRs, with these characteristics collectively indicating a state of profound functional decline.^29^

### 2.3 Metabolic characteristics of Tpex and Tex T cells

Tpex cells are highly adept at generating energy via oxidative phosphorylation (OXPHOS), a process primed by their active engagement of fatty acid oxidation (FAO) pathways.^30^ Their mitochondria are functionally healthy, supporting their long‐term survival and capacity for slow, sustained proliferation.^30^ At the opposite metabolic pole, Tex cells are defined by a glycolytic dependency, which drives mitochondrial dysfunction—a condition marked by rising ROS levels and inefficient ATP production. Furthermore, the accumulation of specific metabolites, such as succinate, can provide feedback regulation on epigenetic enzymes, thereby reinforcing the stability of the exhausted state.^31^, ^32^, ^33^

### 2.4 Transcriptional and epigenetic regulation in Tpex and Tex cells

Our understanding of T‐cell exhaustion has been revolutionised by ATAC‐seq (assay for transposase‐accessible chromatin with sequencing), a technique that maps all open chromatin regions across the genome.^34^ A key insight from these studies is that transcriptional regulation and epigenetic regulation are not independent processes; rather, they form a precise feedback loop that collaboratively shapes the cellular gene expression profile and cellular identity. Open chromatin regions facilitate transcription factor binding and gene activation. Conversely, the binding of transcription factors can recruit specific chromatin‐modifying enzymes responsible for depositing or erasing histone marks (e.g., histone acetyltransferases [HATs] and histone deacetylases [HDACs]), thereby altering the local epigenetic landscape and further reinforcing or modifying the gene expression state.^35^, ^36^

Under conditions of persistent chronic antigen stimulation, exhaustion represents not only a transient decline in T‐cell function but also a distinct, epigenetically fixed state that is largely irreversible during the course of the immune response.^9^, ^37^, ^38^, ^39^ The epigenome of Tex cells undergoes a characteristic reprogramming, involving a concomitant loss of chromatin openness at effector gene loci and a gain at genomic regions housing IR genes.^40^ Anti‐PD‐1 therapy can only transiently revitalise these cells, which ultimately revert to their Tex state.^3^, ^41^ Consequently, targeting the epigenetic modifications that lock in this terminal state is considered a promising direction for improving immunotherapies.

The transcriptional and epigenetic signatures of Tpex cells are defined by openness and plasticity and are orchestrated largely by the key transcription factor TCF‐1. Genomic regions bound by TCF‐1 are enriched with activating histone marks, typified by high levels of H3K4me3 and H3K27ac, thereby maintaining the multipotent differentiation potential of these cells.^21^, ^42^

In contrast, the establishment of a stable ‘epigenetic scar’ most notably characterises the transcriptional programme of Tex cells, a process orchestrated by key factors. This scar manifests as closed chromatin configurations—marked by repressive modifications such as H3K27me3—at the promoters of effector genes (e.g., *Ifng*), while chromatin at exhaustion‐related genes (e.g., *Pdcd1*) remains aberrantly open.^35^, ^43^ In this state, the cells have essentially become irreversibly committed to the dysfunctional fate. Moreover, the Tex state exhibits remarkable stability, persisting even after the antigen is cleared and precluding a return to functional competence.

## 3 THE DIFFERENTIATION PATHWAY FROM Tpex TO Tex CELLS

Understanding the dynamic transition from the Tpex state to terminal exhaustion is crucial, precisely because these states are governed by fundamentally distinct epigenetic landscapes. Recent studies integrating scRNA‐seq and scATAC‐seq data have demonstrated that the differentiation is neither linear nor uniform.^34^ Instead, this pathway branches into multiple trajectories: one leads from the Tpex state to a transitional exhausted state before terminal exhaustion, while another proceeds to an effector‐like exhausted state that ultimately undergoes apoptosis. These branched differentiation trajectories account for the functional heterogeneity observed among exhausted T cells. Intriguingly, in haematologic malignancies such as multiple myeloma, cells displaying a Tex phenotype can maintain their effector capabilities—challenging the conventional view that exhaustion strictly equates to loss of effector capacity.^44^ Collectively, these findings underscore the notable heterogeneity within the exhausted T‐cell pool and suggest an underlying plasticity in cell fate.

The trajectory of this divergent differentiation is predominantly dictated by the strength and duration of T‐cell receptor (TCR) signalling. Sustained exposure to high‐intensity TCR signals promotes the development of exhaustion, with its severity being directly regulated by the intensity and duration of the signals.^45^, ^46^, ^47^ Whether a T cell remains in a multipotent precursor state or commits to an exhausted fate is further determined by a combination of signals from distinct microenvironmental niches and antigen‐presenting cells.^48^ This fate decision is executed through the co‐ordinated actions of transcription factors and epigenetic regulators. The collaborative action of key regulators—NFAT, TOX and NR4A—collectively drives the establishment and maintenance of the exhausted state, while cytokines and metabolites in the microenvironment fine‐tune the function of these factors and the ultimate fate of the cell. Several key immunoregulatory molecules, summarised in Table 1,

**TABLE 1:** Key immune checkpoint molecules and cytokines regulating T cell function.

Category	Molecule name	Ligand	Impact on Tpex/Tex differentiation	Therapeutic implication	Reference
**Co‐stimulatory**	**CD28**	CD80/CD86 (on APCs)	Low signalling: Maintains stem‐like progenitors Strong signalling: Drives effector differentiation	Crucial for PD‐1 blockade efficacy	[^6^]
	**4‐1BB**	4‐1BBL	Promotes expansion of exhausted T cells and their differentiation	Agonists rejuvenate T cells, synergise with checkpoint inhibitors	[^46^]
	**CD27**	CD70	Promotes memory T cell programme, enhances persistence	Enhances CAR‐T cell persistence and function	[^6^]
	**ICOS**	ICOSL	Limits progenitor memory potential; blockade enhances T cell function	Synergistic with PD‐1 inhibition	[^6^]
**Co‐inhibitory**	**PD‐1**	PD‐L1/PD‐L2	Core exhaustion marker; suppresses T cell function	Blockade reinvigorates stem‐like progenitors; immunotherapy cornerstone	[^49^]
	**CTLA‐4**	CD80/CD86	Raises activation threshold, contributes to exhaustion	Combination with PD‐1 inhibitors shows synergy	[^50^]
	**LAG‐3**	MHC class II	Upregulated during differentiation; co‐operates with PD‐1	Combination with PD‐1 inhibitors superior to monotherapy	[^51^]
	**TIM‐3**	Galectin‐9, CEACAM‐1	Marker of Tex T cells	Difficult to rejuvenate; requires combination therapy	[^2^]
	**TIGIT**	CD155 (PVR)/CD112	Promotes immunosuppression and immune evasion	Potential checkpoint target	[^2^]
**Cytokines**	**TGFβ**	TGF‐βR	Suppresses T cell function Maintains stem‐like progenitor pool	Target requires balancing dual roles	[^52^]
	**IL‐2**	IL‐2R (CD25)	Controlled: Drives effector differentiation Prolonged: Drives terminal exhaustion PD‐1 suppresses IL‐2 production	Requires fine‐tuning to balance differentiation/stemness	[^53^]
	**IL‐10**	IL‐10R	Context‐dependent: Chronic infection: Suppressive Solid tumours: Stimulates CD8+ T cells Blood cancers: Supports progenitors	Potential antagonist or agonist, depending on context	[^54^,^55^]
	**IL‐21**	IL‐21R	Supports effector differentiation while delaying terminal exhaustion	Promotes potent, durable T cell responses	[^56^]
	**IL‐12**	IL‐12R	Short‐term: Enhances effector function Sustained: Drives terminal exhaustion, impairs stemness	Requires precise therapeutic sequencing	[^57^]
	**Type I interferon**	IFNAR	Drives exhaustion by promoting terminal differentiation and reducing stemness	A promising target for rational combination therapy.	[^58^]

*Note*: This table is a synthetic summary. All information presented (including molecular categories, functional impacts and therapeutic implications) was synthesised from the original research papers cited in the ‘Reference’ column for each respective row. These original papers provide detailed evidence, including experimental validation and data analysis. The work of this review did not involve the re‐analysis of raw data from public repositories such as GEO or TCGA.

**FIGURE 1:**
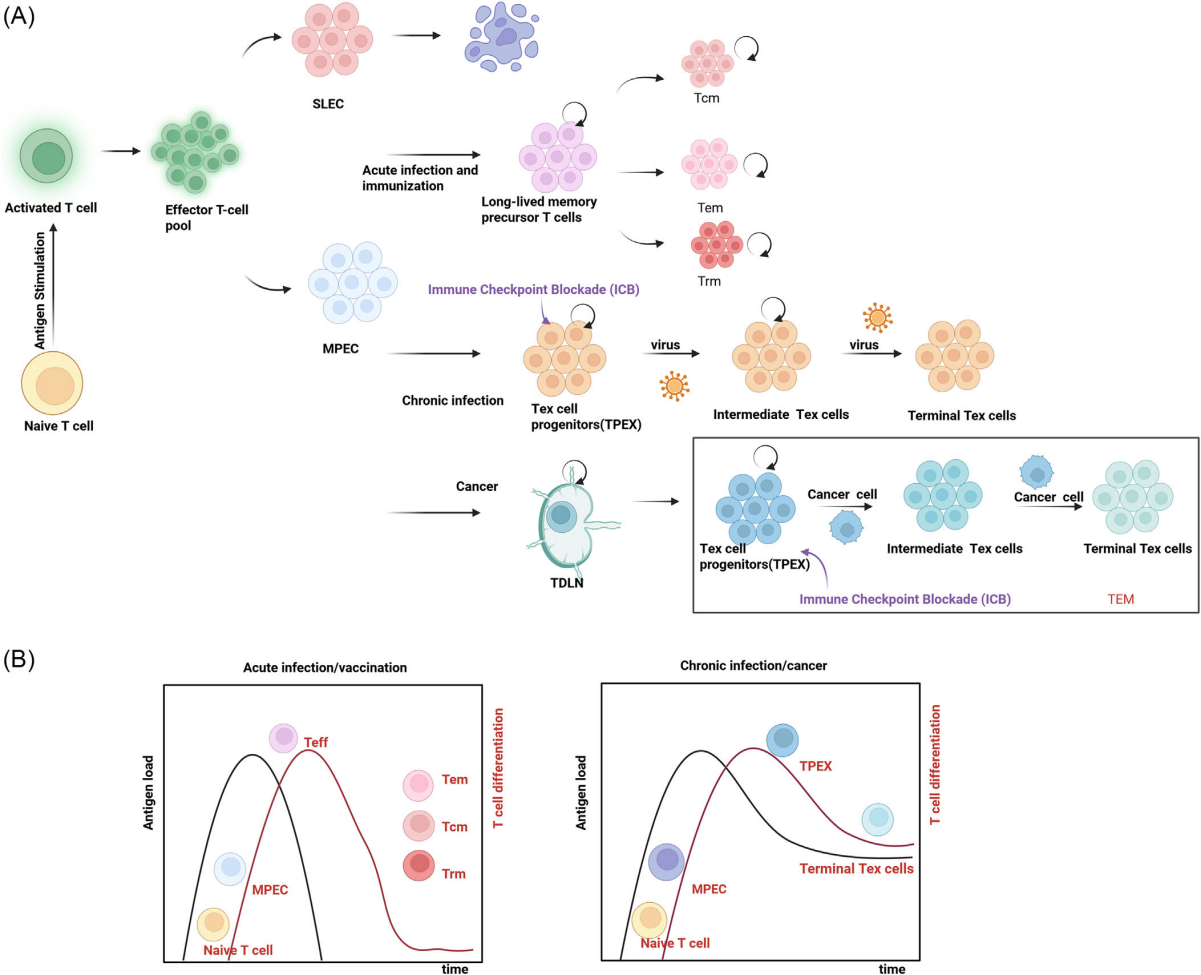
Fate decisions of antigen‐specific CD8^+^ T cells under acute versus chronic antigen exposure. (A) Schematic overview of differentiation trajectories. (B) Antigen kinetics and corresponding T cell subset dynamics. This figure was created using BioRender.com.

Play instrumental roles in this process. Overall, the exhausted T‐cell compartment is best understood not as a uniform entity, but as a hierarchically organised system. This system's architecture is established early, beginning with the antigen‐driven divergence of naïve T cells into distinct precursor lineages: memory precursor effector cells (MPECs) and short‐lived effector cells (SLECs).^2^ Following this initial fate split, the specific tissue microenvironment exerts a defining influence on the further differentiation of these progenitor populations^11^, ^12^, ^59^ (Figure 1).

### 3.1 T‐cell differentiation in acute infection and vaccination

The encounter with a pathogen during acute infection triggers robust activation and extensive clonal expansion of antigen‐specific naïve T cells. This expanded population gives rise to SLECs. This lineage is characterised by potent cytotoxicity and the capacity to secrete effector molecules such as interferon‐γ and tumour necrosis factor, which together facilitate the rapid elimination of infected cells (Figure 1). Following pathogen clearance and the cessation of antigenic signals, most effector T cells are removed via apoptosis to preserve immune homeostasis.^2^ A minor fraction, however, escapes this fate and commits to the memory T‐cell (Tmem) lineage.^2^ This long‐lived memory compartment, comprising central, effector and tissue‐resident subsets, persists in the host. Upon re‐exposure to the same pathogen, these cells mediate an accelerated and more vigorous secondary immune response^2^ (Figure 1).

### 3.2 T‐cell exhaustion in chronic infection and cancer

In chronic infections and cancer, the immune system's failure to resolve antigens results in sustained stimulation and recurrent proliferation of antigen‐specific naïve T cells. Counterintuitively, this persistent antigenic and inflammatory signalling prevents MPECs from maturing into fully functional effectors (Figure 1). Instead, they acquire an exhausted state. This condition is defined by a progressive differentiation pathway that originates from stem‐like progenitor exhausted T (Tpex) cells and culminates in Tex cells.^29^, ^60^, ^61^ Within this hierarchy, the majority of progenitor cells remain in a relatively quiescent state. Upon demand, these quiescent progenitor exhausted T cells can give rise to a CX3CR1^+^, natural killer cell receptor‐expressing effector‐like intermediate population, which possesses potent cytotoxic and proliferative capacities. When stimulated, these quiescent Tpex cells can repeatedly give rise to effector‐like intermediate exhausted T cells. This intermediate population expresses markers like CX3CR1 and various natural killer cell receptors, and develops strong cytotoxic and proliferative functions.^62^, ^63^ Evidence points to tumour‐draining lymph nodes (TDLNs) as a critical site where commitment to exhaustion is often initiated. Here, T cells are primed by tumour antigens and start to display early signs of exhaustion. The TCF1^+^ progenitor T cells localised in TDLNs display key stem‐like attributes, including pronounced self‐renewal ability and phenotypic plasticity (Figure 1). Following their migration into the immunosuppressive tumour microenvironment (TME), relentless antigen encounter combined with inhibitory signals promotes the swift adoption of a terminal exhaustion gene programme. This transition ultimately leads to the generation of dysfunctional, Tex cells^6^, ^61^ (Figure 1). Critically, prolonged stimulation (e.g., for more than ∼30 days) drives extensive epigenetic remodelling, culminating in a fixed epigenetic scar that stabilises the exhausted state. This fixation renders the exhausted state largely irreversible, meaning that even transient relief from inhibition (e.g., via PD‐1 blockade) cannot fully restore normal cellular function.^3^ Consequently, the persistence of pathogens or malignancies is associated with a gradual erosion of T‐cell functionality and eventual collapse of the immune response (Figure 1).

Notably, emerging data suggest that T cells displaying a profoundly exhausted phenotype may, in some contexts, maintain residual effector activity, challenging the notion that exhaustion equates to a complete loss of function.^64^ Therefore, the exhausted T‐cell pool is best understood as a complex system comprising cells in multiple states, including Tpex, intermediate/transitional and Tex subsets (Figure 1).

## 4 THE DIFFERENTIATION REGULATORY NETWORK FROM A MULTIOMICS PERSPECTIVE

The progression of T cells from a Tpex progenitor state to terminal exhaustion is not a linear cascade but a dynamic, self‐reinforcing process orchestrated by an integrated multiomics network. This network operates across transcriptional, epigenetic, metabolic and posttranslational layers, with each layer both responding to and actively shaping the others. The initial fate bias (stemness vs. exhaustion) is established by transcriptional reprogramming. These transcriptional commands are then epigenetically cemented through histone and DNA modifications, creating a stable cellular memory. Concurrently, metabolic reprogramming meets the demand for energy and biosynthetic precursors, which are critical to drive this transition, while its byproducts act as signalling molecules to directly modulate the expression of epigenetic enzymes, thereby linking environmental cues to gene expression. Finally, posttranslational modifications provide rapid, fine‐tuning adjustments to protein function, generating and stabilising the new cellular state. In this chapter, this network is dissected layer by layer, emphasising the interconnectedness of the layers in driving the irreversible commitment to exhaustion.

### 4.1 Transcriptomic layer: The dynamic evolution of the core transcription factor network

A multilayered and dynamically evolving network of transcription factors—delineated by transcriptomic studies—governs the stepwise progression.

#### 4.1.1 Guardians of the stemness programme: TCF‐1 and its collaborative network

**FIGURE 2:**
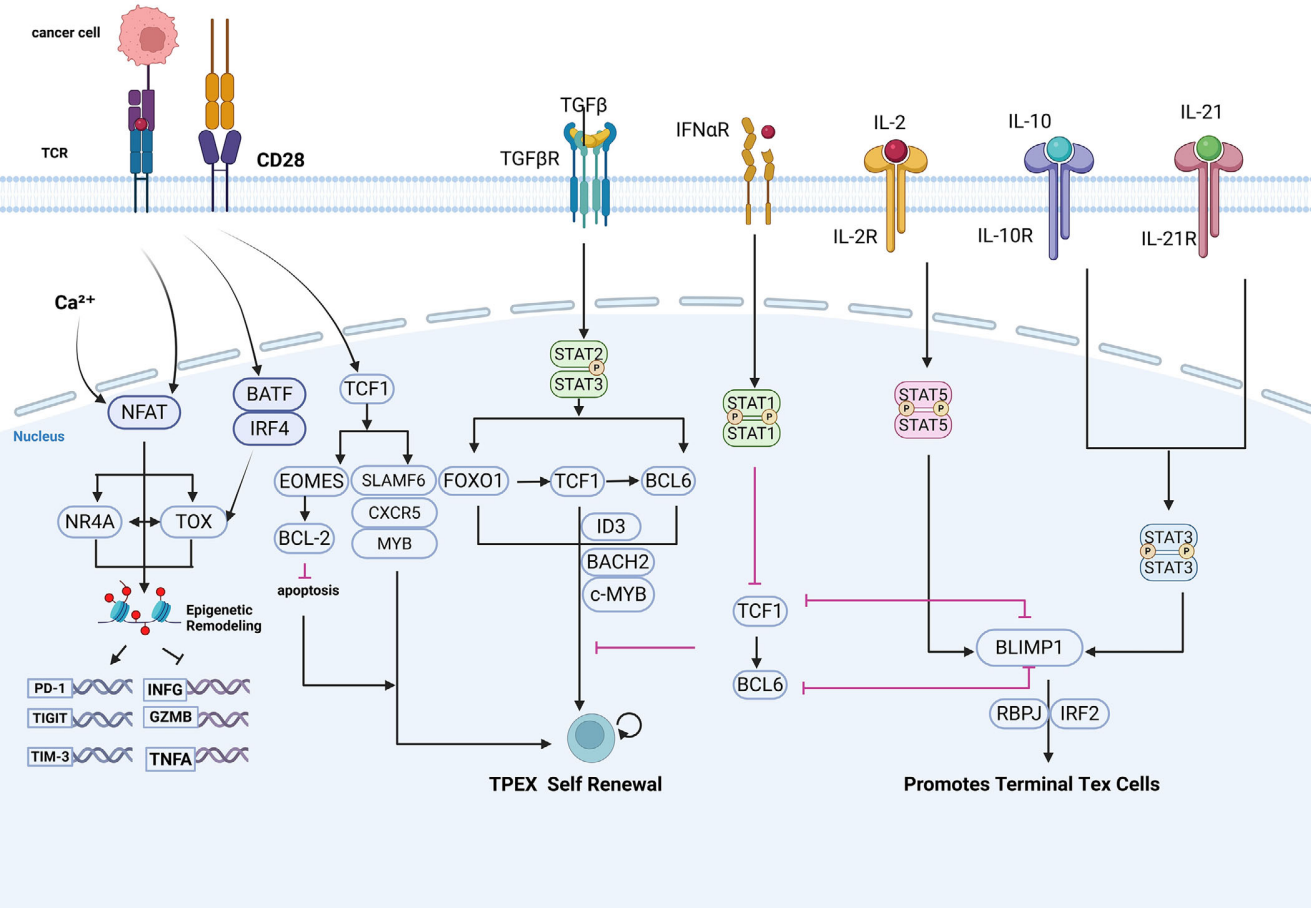
Transcriptional networks and microenvironmental signals regulating T cell exhaustion. This figure was created using BioRender.com.

TCF‐1 serves as the master transcriptional regulator that defines and sustains the identity of precursor exhausted T (Tpex) cells.^65^ It guides Tpex cell fate through a dual regulatory mechanism: the transcriptional repression of the terminal effector factor T‐bet and the concomitant induction of the pro‐survival, memory‐like factor Eomes^14^, ^66^ (Figure 2). Moreover, TCF‐1 directly inhibits the expression of key drivers of terminal exhaustion, such as TOX and Prdm1, thereby preserving cellular plasticity (Figure 2). At the level of target gene regulation, TCF‐1 activates the transcription of its encoding gene (*Tcf7*) and its cofactor *LEF1*, forming an essential positive feedback loop. It also upregulates genes that support stem‐like features, including Slamf6, *Cxcr5*, *Myb* and Bcl2^65^ (Figure 2). This transcriptional programme is reinforced by a co‐operative network involving LEF1, MYB and ID3. MYB functions in concert with TCF‐1 to jointly consolidate the stemness circuitry of Tpex cells, ensuring their long‐term persistence.^67^ ID3 helps maintain the ‘stemness’ and proliferative potential of T cells by inhibiting E protein activity.^68^ LEF1 functions as a cofactor for TCF‐1, binding DNA co‐operatively and increasing the transcription of downstream genes in the Wnt signalling pathway.^13^

**FIGURE 3:**
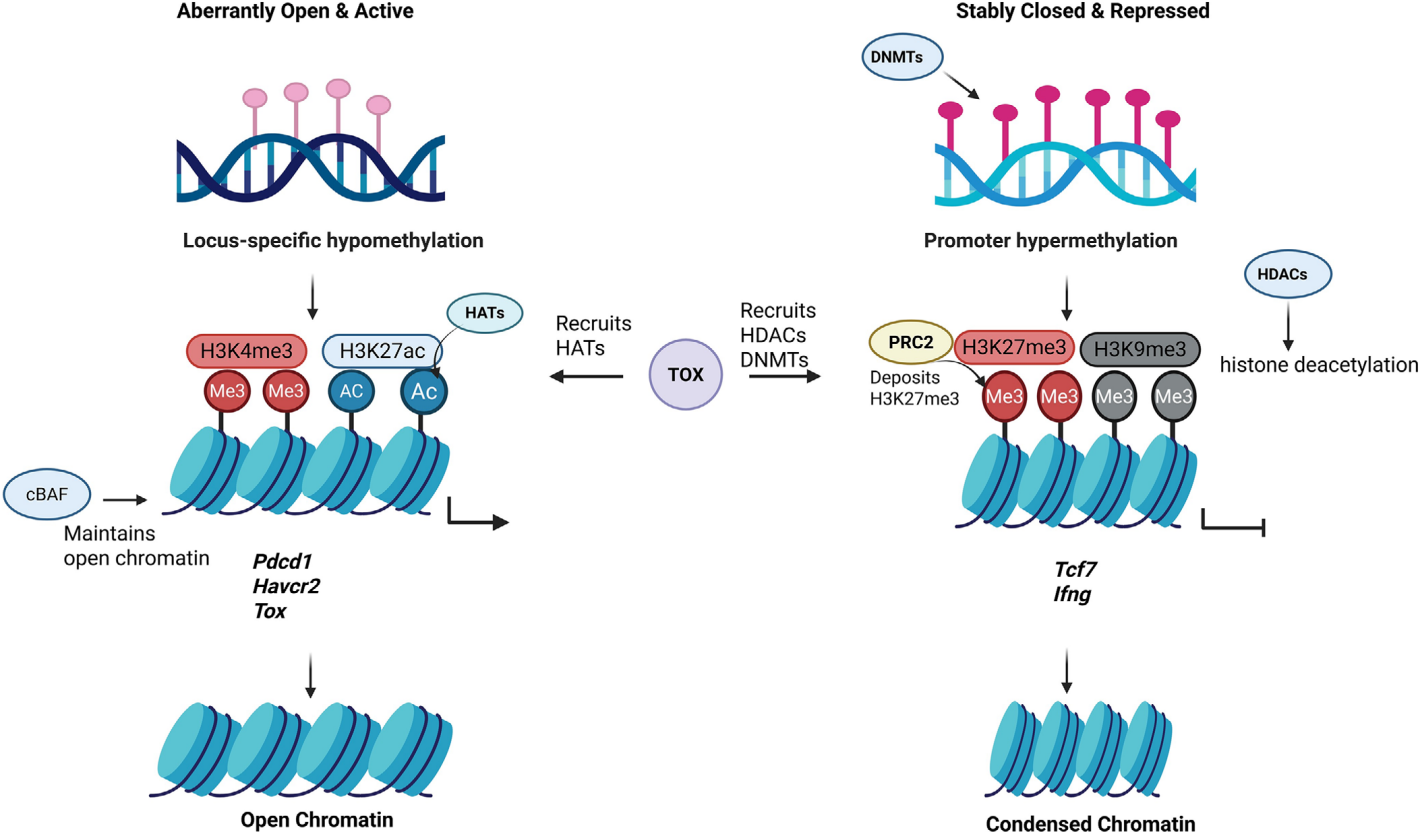
TOX‐mediated establishment of the epigenetic scar in terminally exhausted T cells. This figure was created using BioRender.com.

At the epigenetic level, TCF‐1 recruits coactivators such as p300/CBP to its target loci, depositing active histone marks such as H3K4me3 and H3K27ac, which collectively establish an open and plastic chromatin landscape^69^, ^70^ (Figure 3). Functionally, TCF‐1 loss‐of‐function experiments have demonstrated that its ablation in Tex cells results in the complete loss of their ability to proliferate and severely impairs core immune functions, whereas its overexpression can increase cytokine production and reduce co‐IR expression.^65^

In summary, by integrating upstream signals, TCF‐1 functions as the central orchestrator of a multilayered regulatory system encompassing transcriptional control, co‐operative factor networks and epigenetic remodelling. This integrated action is essential for establishing and maintaining the Tpex cell identity, thereby preserving a pool of precursors with functional plasticity.

#### 4.1.2 Activation and core drivers of the exhaustion programme: TOX and its synergistic partner NR4A

The transcriptional foundation of T cell exhaustion is laid by factors antagonistic to the TCF‐1‐dependent stemness axis. This process is initiated by the disruption of a key transcriptional partnership. Upon initial T cell activation, the transcription factor NFAT enters the nucleus, where its partnership with AP‐1 is essential for driving a productive effector gene programme^71^ (Figure 2). However, under the duress of chronic stimulation, this co‐operative balance fails. AP‐1 availability wanes while NFAT activity persists, creating a surplus of nuclear NFAT that operates without its canonical partner.^72^ In this unopposed state, NFAT directly transactivates genes central to the exhaustion programme, including the IR PD‐1 (via its Pdcd1 promoter) and the transcription factors NR4A and TOX.^73^ The net effect of this NFAT dominance is a transcriptional shift away from effector functions and towards a state pre‐programmed for exhaustion, characterised by the upregulation of inhibitory pathways (Figure 2).

Sustained TCR signalling, a hallmark of chronic infection and cancer, acts as the primary trigger for the rapid and persistent induction of TOX. This induction is mechanistically rooted in the NFAT activation cascade downstream of the TCR. Once expressed, TOX executes a feedback loop that consolidates the exhausted fate, notably by further amplifying the expression of PD‐1 and other co‐IRs, thereby cementing the dysfunctional phenotype of early exhausted T cells.^43^, ^73^, ^74^

The role of TOX is fundamental and non‐substitutable in both launching and perpetuating the Tex state. Functioning as a master epigenetic architect, TOX possesses pioneer factor activity. It invades compacted chromatin domains at loci critical for exhaustion (such as *Pdcd1* and *Havcr2*), orchestrates the recruitment of chromatin‐modifying complexes like HATs and catalyses the opening of these regions to facilitate gene transcription^43^, ^73^, ^74^ (Figure 2). Its action is potentiated by the transcription factor Eomes, propelling T cells into deeper dysfunction.^12^ Through its sustained expression, TOX guides the formation of an exhaustion‐associated epigenetic landscape (e.g., specific histone acetylation patterns), ultimately leading to an epigenetic scar that locks the cell in the Tex state. It also indirectly suppresses the transcription of *Tcf7* (encoding TCF‐1), promoting attenuation of the stem‐like programme.

NR4A transcription factors contribute to T‐cell exhaustion through two primary mechanisms: co‐operating with TOX and repressing effector function. The calcium influx triggered by TCR activation rapidly induces high‐level transcription of NR4A family members. NR4A proteins subsequently act as transcription factors to further regulate downstream target genes^73^ (Figure 2). Persistent TCR signalling drives NR4A proteins to accumulate and be retained within the nucleus. Acting synergistically with TOX, NR4A binds co‐operatively with TOX to a large number of genomic sites, potently coactivating the transcription of exhaustion‐related genes^73^ (Figure 2). Additionally, this functional suppression is achieved through direct interference with signalling pathways (e.g., AP‐1/NF‐κB), leading to the downregulation of effector cytokine genes, including IFN‐γ.^8^, ^19^, ^29^, ^75^

#### 4.1.3 Coregulators of the exhaustion programme: The BATF/IRF4 complex and BLIMP‐1

BATF and IRF4 are upregulated during the exhaustion differentiation programme and form a transcriptional complex.^76^ Mechanistically, this complex assumes the role of canonical AP‐1 factors (e.g., FOS/JUN) by occupying their genomic binding sites. Functionally, it drives the expression of IRs like PD‐1 and contributes to the suppression of effector responses. The BATF/IRF4 complex also acts synergistically with TOX and is a key factor in establishing the initial exhaustion programme.^77^, ^78^, ^79^

BLIMP‐1 (encoded by the *Prdm1* gene) directly suppresses the Tpex programme and promotes cellular exhaustion.^80^ It employs a dual mechanism to suppress stemness: directly repressing the *Tcf7* (TCF‐1) promoter and inhibiting BCL6 expression ^81^, ^82^ (Figure 2). Together, these actions actively eliminate the stem‐like potential of cells. Simultaneously, BLIMP‐1 can promote the expression of certain exhaustion‐associated proteins, pushing the cell towards a terminal effector state.^81^ The BLIMP‐1 protein targets the *Tcf7* gene promoter and recruits HDACs and histone methyltransferases (HMTs). This recruitment condenses the local chromatin environment, a shift marked by H3K27 deacetylation and the deposition of repressive marks like H3K9me3. This compaction potently suppresses TCF‐1 transcription. Furthermore, BLIMP‐1 represses key genes essential for maintaining Tpex cell self‐renewal, metabolism and survival, including *Slamf6* and *Myc*.

Thus, the antagonism between TCF‐1 and TOX not only defines the initial transcriptional fate but also initiates downstream epigenetic and metabolic reprogramming, setting in motion the self‐reinforcing cycle that leads to the multilayered ‘lock‐in’ characteristic of exhaustion.

In summary, CD8^+^ T‐cell exhaustion arises from a dynamically balanced transcriptional tug‐of‐war between opposing regulatory programmes. The TCF‐1‐driven stemness and plasticity programme competes with the exhaustionlocking programme initiated by TOX and reinforced by collaborators such as NR4A and BATF/IRF4, whereas BLIMP1 further tilts the balance by actively suppressing the stemness circuitry. This core transcriptional conflict triggers a downstream cascade of regulatory events coupled with progressive epigenetic remodelling—shifting the open, plastic chromatin state maintained by TCF1 towards a stable, repressive epigenetic scar orchestrated by TOX, BLIMP1 and other factors. Together, these interconnected layers of the multiomics network drive an irreversible transition, committing T cells to differentiate from a multipotent precursor state to a functionally fixed Tex state.

### 4.2 The epigenetic layer: The 'memory' and locking of cell fate

The transcriptional decisions described above are consolidated and made heritable through extensive epigenetic reprogramming, a process that is itself sensitive to the metabolic milieu of the cell. The epigenetic reprogramming that underpins T‐cell exhaustion chiefly manifests through four interconnected domains: altered chromatin accessibility, shifts in DNA methylation patterns, dynamic histone modifications and comprehensive chromatin remodelling (Figure 3).

#### 4.2.1 Chromatin accessibility

Chromatin accessibility, denoting the physical openness of genomic DNA, determines its availability for transcription factor binding. In Tpex cells, key genes governing stemness, self‐renewal and multipotency (e.g., *Tcf7*, *Slamf6*) reside within regions of highly accessible chromatin. The transcription factor TCF‐1 sustains this permissive state by binding to enhancers of these genes and recruiting remodellers like the PBAF (SWI/SNF) complex, which in turn facilitates further TCF‐1 engagement.^83^, ^84^ Conversely, Tex cells possess a globally distinct epigenetic landscape compared to effector and memory CD8^+^ T cells. A hallmark of terminal exhaustion is increased accessibility at exhaustion‐specific enhancers (e.g., those associated with the *PDCD1* gene), leading to high expression of IRs, coupled with decreased accessibility at enhancers of effector genes (e.g., *Ifng*), resulting in loss of effector function^40^ (Figure 3).

#### 4.2.2 Histone modifications

Histone acetylation promotes a transition to a more open, permissive chromatin state. This relaxed structure enhances transcription factor access and thereby potently upregulates gene expression. Conversely, histone methylation can hinder transcription factor binding and suppress transcription. In Tpex cells, the activating histone marks H3K4me3 and H3K27ac prominently mark the regulatory regions of genes involved in stemness and memory, such as *Tcf7*. This dual activation signal helps maintain the expression of these genes, thereby supporting cellular plasticity and multipotency.^42^ In stark contrast, Tex cells undergo profound epigenetic reprogramming. A repressive epigenetic state, marked by H3K27me3 deposition, is acquired at the promoters of effector genes like *Ifng* and *Tnf*, leading to chromatin compaction and silencing. In contrast, the enhancers of exhaustion‐linked genes (e.g., *Pdcd1* and *Tox*) are decorated with activating marks such as H3K27ac, rendering the chromatin abnormally open and transcriptionally active^85^ (Figure 3). Furthermore, key stemness gene loci may acquire additional repressive marks, such as H3K9me3, resulting in more complete and stable gene silencing.

#### 4.2.3 DNA methylation

DNA methylation can physically impede transcription factors from recognising and binding their target DNA sequences. Furthermore, it can promote the recruitment of corepressor complexes (e.g., those containing HDACs) to the region, altering the chromatin structure towards a more condensed state (heterochromatin), ultimately leading to gene silencing.^13^, ^62^, ^86^ In Tex cells, DNA methylation patterns diverge at specific loci: promoters of stemness‐related genes like Tcf7and Il2become hypermethylated, while those of exhaustion‐linked genes such as *Pdcd1* tend to be hypomethylated. This distinct methylation profile contributes to the epigenetic scar that helps fix the exhausted state in an irreversible manner. Mechanistically, DNA methylation‐mediated reprogramming of *Runx3* has been shown to act as a key regulator of CD8^+^ T‐cell function. Reversing methylation at the *Runx3* promoter enhances the infiltration of CD8^+^ tumour‐infiltrating lymphocytes and mitigates T‐cell exhaustion.^87^

#### 4.2.4 Chromatin remodelling

The chromatin remodelling complex SWI/SNF critically regulates chromatin architecture and gene expression in CD8^+^ T cells through its distinct subcomplexes. The cBAF subcomplex drives effector and terminal differentiation programmes. Disruption of cBAF enhances stemness and persistence in these cells while suppressing terminal exhaustion^88^, ^89^, ^90^ (Figure 3). In contrast, the PBAF subcomplex restrains the differentiation and expansion of effector‐like exhausted T cells. It does so by regulating the accessibility of TCF1 binding sites, thereby facilitating TCF1 occupancy and helping to maintain a quiescent precursor state. Therefore, inhibiting PBAF acts as a key modification that unlocks a superior functional state in exhausted T cells, driving them towards a CX3CR1^+^ effector‐like phenotype. These reprogrammed cells are endowed with greater proliferative potential and secretory function, which directly translates into more effective tumour control and augments the benefit obtained from ICB.^84^, ^91^, ^92^

As a central orchestrator, TOX directs this epigenetic reprogramming. Context‐dependent recruitment of cofactors enables TOX to assemble activating complexes (e.g., containing HATs) to promote a transition to an open chromatin state at exhaustion‐related loci. Conversely, it can recruit repressive complexes containing HDACs and DNA methyltransferases (DNMTs) to silence stemness and effector genes, which collectively mediate the restructuring of chromatin towards a locked conformation.^93^

Consequently, the epigenetic scar functions as an active regulatory entity rather than a mere passive marker. It locks the transcriptional programme of exhaustion‐associated genes—a process reinforced by metabolic inputs—and actively drives the irreversible fixation of the cellular state.

In summary, this dynamic process—spanning Tpex cells to Tex cells—involves a progression from cellular plasticity to phenotypic fixation. Understanding this process provides a rationale for developing epigenetic therapeutic strategies aimed at enhancing immunotherapy efficacy. These strategies can be achieved by erasing parts of the epigenetic scar with agents like HDAC or DNMT inhibitors, or by using genetic engineering to reactivate stemness programmes (e.g., those controlled by TCF‐1) in Tex cells to reverse their dysfunctional state.^93^

### 4.3 The metabolomic layer: Metabolic reprogramming drives cell fate transitions

**FIGURE 4:**
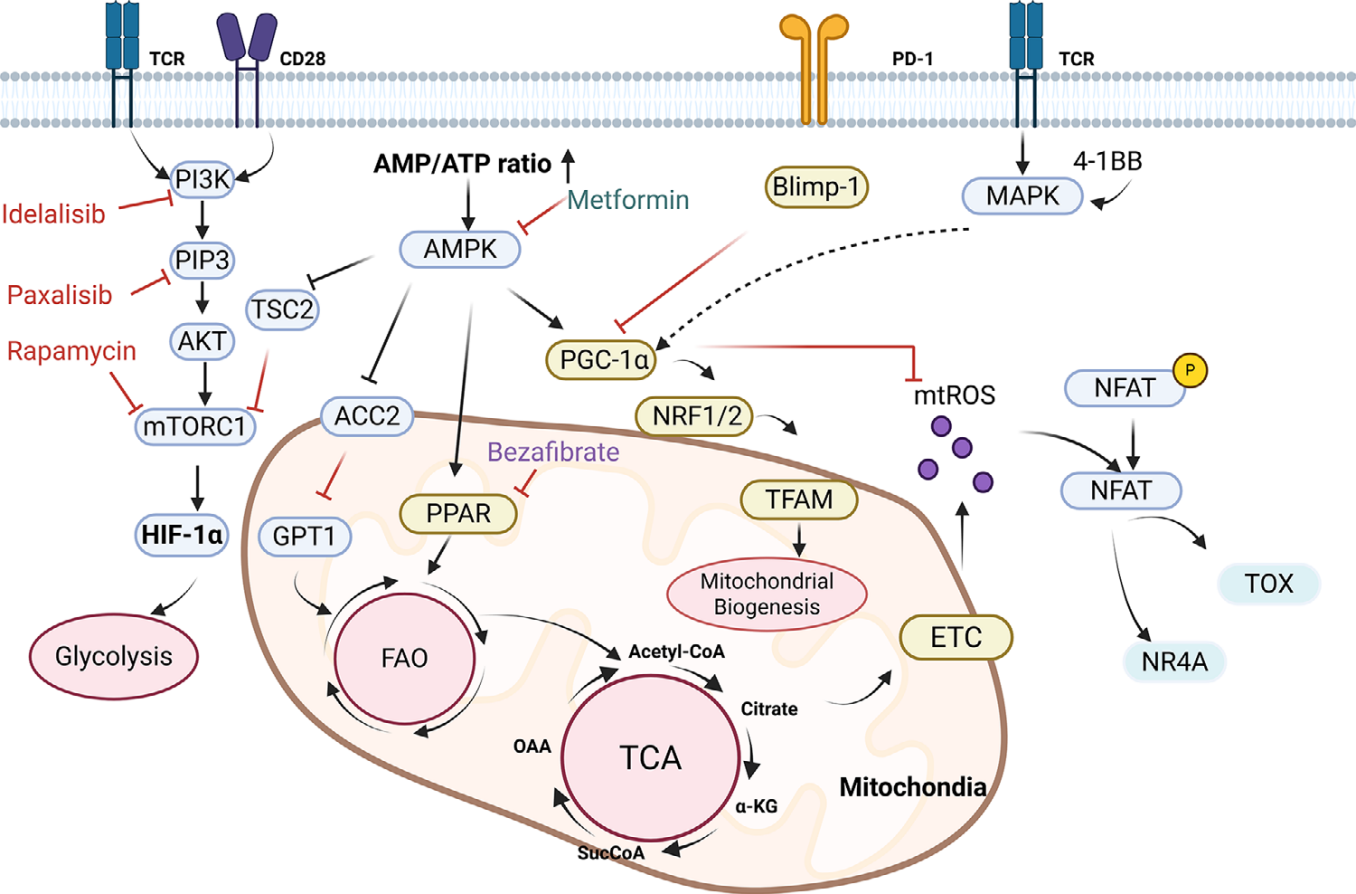
Metabolic reprogramming from oxidative phosphorylation to aerobic glycolysis during T‐cell exhaustion. This figure was created using BioRender.com.

The functional divergence between the progenitor and terminal states is not only driven by distinct transcriptional and epigenetic programmes but also fundamentally rooted in the opposing metabolic states in these cell types. Metabolic reprogramming is continuously active throughout exhaustion differentiation and is manifested primarily across three interrelated dimensions: (a) a switch in core energy metabolism programmes; (b) the activation of key metabolic sensing and regulatory pathways; and (c) feedback regulation of cell fate by metabolites acting as signalling molecules (Figure 4). This reprogramming serves a dual role: it fulfils the distinct energy requirements of these cells while actively moulding and cementing their commitment to specific fates.

#### 4.3.1 The switch in core energy metabolism programmes

Tpex and Tex cells employ fundamentally different energy production strategies, which directly determine their persistence and function. Metabolic programming in Tpex cells is centred on mitochondrial health and oxidative metabolism. To support their long‐term survival, slow proliferation and self‐renewal potential, Tpex cells utilise OXPHOS and FAO as primary energy sources.^30^ This metabolism provides ample ATP while supporting robust mitochondrial function and maintaining ROS at physiological levels. Orchestrating this metabolic state is PGC‐1α, a master co‐ordinator of mitochondrial biogenesis and quality control. PGC‐1α exerts its effects by activating nuclear respiratory factor 1 (NRF1), thereby regulating nuclear genes for mitochondrial components, and by upregulating mitochondrial transcription factor A (TFAM), which is essential for mitochondrial DNA maintenance and gene expression. In summary, PGC‐1α provides the molecular foundation for efficient OXPHOS.

In contrast, Tex cells undergo a metabolic reprogramming characterised by a stable reliance on aerobic glycolysis. This glycolytic dependency results in inefficient ATP generation, creating an energy deficit that cannot support robust effector functions (Figure 4). Concomitantly, their mitochondria exhibit profound dysfunction, marked by diminished mass, dissipated membrane potential, fragmented networks and elevated ROS production. This metabolic shift directly contributes to the failure of effector functions.

#### 4.3.2 Activation of key metabolic sensing and regulatory pathways

The aforementioned metabolic switch is driven primarily by signals from the TME and persistent TCR signalling through two central pathways.

The hypoxic conditions typical of chronic infections and tumours serve as a critical driver of T‐cell exhaustion. This is mediated primarily by hypoxia‐inducible factors (HIFs, especially HIF‐1α) and associated epigenetic remodelling.^75^, ^94^ A key downstream effect of HIF‐1α is the metabolic reprogramming of T cells: it elevates glucose uptake via upregulation of GLUT1 and enhances glycolytic flux by increasing the expression of glycolytic enzymes^95^, ^96^, ^97^ (Figure 4). Through the hypoxia–HIF‐1α axis, immune effector genes are silenced via epigenetic mechanisms mediated by HDAC1 and PRC2.^98^ In the TME, hypoxia‐driven HIF‐1α signalling elevates the expression of CD39 and CD73 on intratumoural Tregs and exhausted CD8^+^T cells. This elevation ultimately results in heightened accumulation of extracellular adenosine.^99^ Adenosine signalling through the A_2_A receptor inhibits T‐cell activity, reinforces exhaustion and impairs antitumour immunity. Concurrently, hypoxia downregulates PGC‐1α. Therefore, a strategy to enhance the anti‐tumour potency of tumour‐specific CD8^+^ T cells is to reintroduce PGC‐1α, thereby restoring their metabolic health and effector capabilities^48^, ^100^ (Figure 4).

Chronic TCR signalling leads to persistent PI3K–AKT signalling, which strongly activates mTORC1^101^ (Figure 4). Acting as a metabolic master switch, mTORC1 orchestrates a profound reprogramming: it drives the transcription of glycolytic genes (including GLUT1 and HK2), stimulates ribosome biogenesis and protein synthesis and concurrently inhibits autophagy and OXPHOS. This state is reinforced as constitutively active mTORC1 elevates key transcription factors like c‐Myc and HIF‐1α, which potently activate the glycolytic programme, effectively locking exhausted T cells into a high‐dependency glycolytic state. However, persistent mTOR signalling negatively regulates mitochondrial biogenesis and quality control, leading to a loss of OXPHOS capacity. This leaves exhausted T cells inefficient in catabolising fatty acids and amino acids through oxidative pathways for ATP generation.^61^ The resulting accumulation of ROS—generated primarily by dysfunctional mitochondria—damages DNA and proteins and activates stress signalling pathways, further exacerbating the exhaustion state^102^ (Figure 4).

#### 4.3.3 Feedback regulation of cell fate by metabolites as signalling molecules

The accumulation of specific metabolites drives epigenetic changes that ultimately promote T‐cell exhaustion. Elevated α‐ketoglutarate (α‐KG) serves as a key cofactor for demethylases like TET and KDM, thereby sustaining an open, plastic chromatin landscape that permits the transcription of stemness‐associated genes, including *Tcf7*.^103^ In contrast, the accumulation of inhibitory metabolites such as succinate inhibits demethylase activity, leading to hypermethylation (both DNA and histone methylation, e.g., H3K27me3). This process contributes to the formation of the epigenetic scar, silences effector genes (e.g., *Ifng*) and locks in the exhaustion programme.^104^ Furthermore, dysfunctional mitochondria produce excessive amounts of mitochondrial ROS (mtROS), which drives the exhausted state through a dual action: the upregulation of IRs and the repression of genes essential for effector function.^102^

Critically, these findings underscore the idea that the metabolic shift from OXPHOS to glycolysis is not merely an energy adaptation. As exemplified by the effects of succinate, the resulting metabolic reprogramming directly shapes the repressive epigenetic landscape (e.g., via H3K27me3 deposition) that silences effector genes, constituting a core means by which metabolic reprogramming epigenetically locks in the exhausted state (as detailed in Section 4.2.2).

In summary, the transition from Tpex to Tex cells involves a fundamental metabolic reprogramming, co‐ordinately driven by the HIF‐1α and mTORC1 pathways, which switches the cellular energy hub from oxidative metabolism towards glycolytic dependency. This reprogramming fuel a self‐reinforcing circuit: metabolic byproducts act to consolidate the exhaustion‐associated epigenetic and transcriptional signature, which in turn fixes the dysfunctional metabolic state. This transition is co‐ordinately driven by the HIF‐1α and mTORC1 pathways. The resulting metabolites actively feed back into the epigenetic and signalling landscape to reinforce the exhaustion programme, creating a self‐perpetuating vicious cycle that irreversibly commits the cell to an exhausted fate. Therefore, targeting metabolic reprogramming is a promising therapeutic strategy. Interventions like PGC‐1α agonists, aimed at enhancing mitochondrial function, serve dual purposes: reinvigorating T‐cell bioenergetics and interrupting the self‐perpetuating metabolic–epigenetic feedback loop. This dual action could potentially reverse the locked state of exhaustion, thus forming a strong basis for novel combination therapies designed to restore T‐cell function.

### 4.4 The proteomic and PTMs layer: Pathway regulation at the functional execution level

#### 4.4.1 Protein expression profiling

**FIGURE 5:**
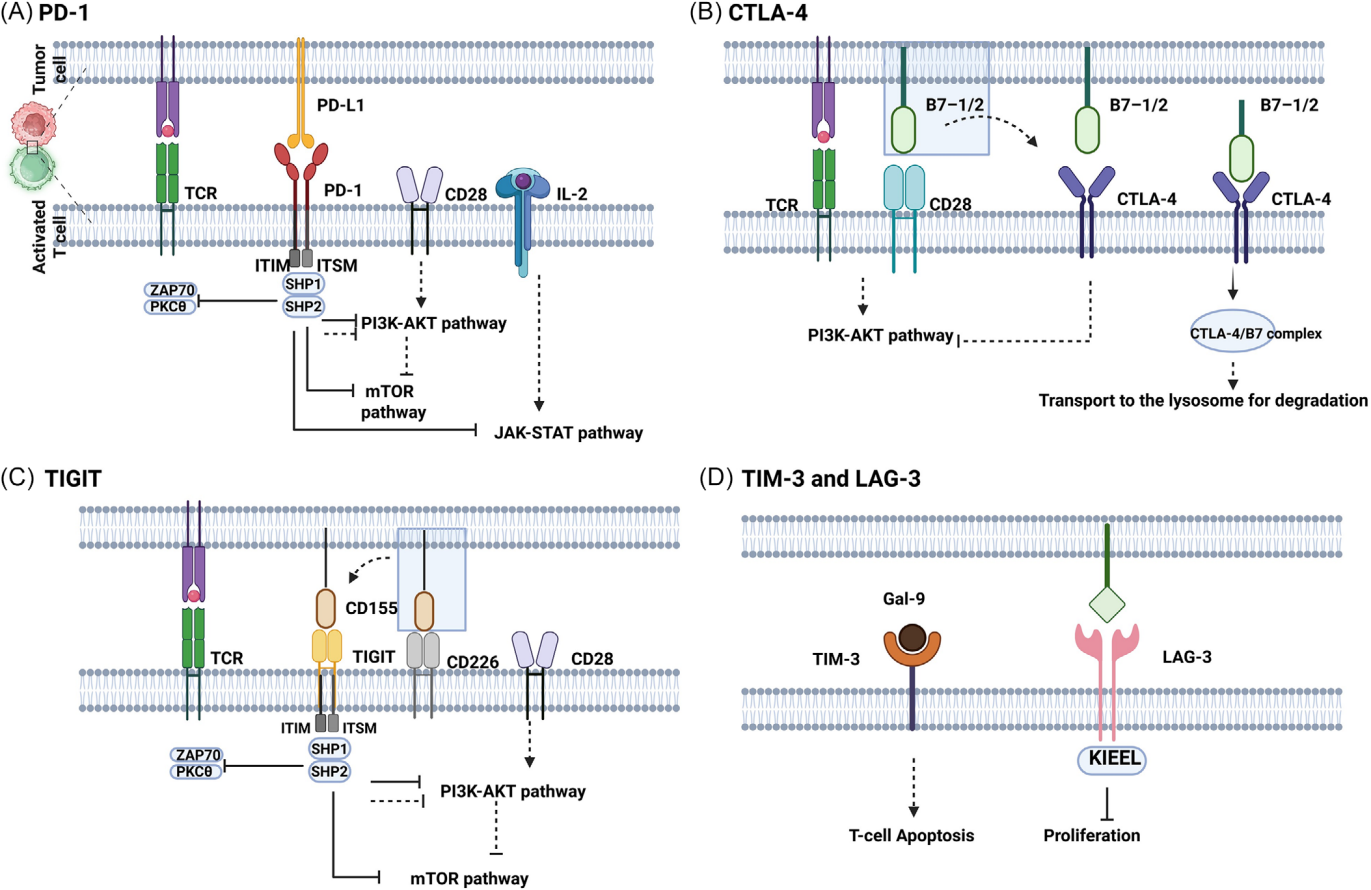
Mechanisms of inhibitory receptor signalling in T‐cell exhaustion. This figure was created using BioRender.com.

Comparative proteomics has uncovered stark differences in protein composition and abundance distinguishing Tpex cells from their Tex counterparts, differences that directly govern their divergent functional identities. In Tex cells, the balance of key TCR signalling proteins shifts: effector proteins like ZAP70 are downregulated, while inhibitory proteins such as SHP2 are elevated.^105^, ^106^ Functional impairment in exhausted T cells arises from alterations at multiple signalling levels. First, the levels of key effector proteins—including TNF‐α, perforin and granzymes—are diminished.^2^ Second, altered abundance and function of receptors for cytokines like IL‐2, IL‐12 and IL‐21 result in dysregulated STAT pathway activity, further promoting exhaustion^6^ (Figure 2). Additionally, TGF‐β signalling via the SMAD pathway elevates the expression of IRs such as PD‐1 and TIM‐3 (Figure 2). IL‐10 contributes to an immunosuppressive microenvironment, indirectly preventing effective antigen clearance by T cells and thereby driving them towards exhaustion^107^ (Figure 2). A central proteomic shift involves the replacement of stemness‐maintaining transcription factors (e.g., TCF‐1) with exhaustion‐driving factors (e.g., TOX, NR4A, BLIMP‐1).^73^ This transcriptional reprogramming directly drives a concomitant shift in the cell surface proteome from a profile marked by CXCR5 to one dominated by robust co‐expression of multiple checkpoint proteins, notably PD‐1, TIM‐3 and LAG‐3^23^ (Figure 5). Key functional outputs are severely compromised: the expression and function of effector molecules and signalling pathway components are impaired, leading directly to the loss of effector function and attenuated responses to stimulation. Furthermore, metabolic reprogramming is evident at the translational level, with a shift from OXPHOS complexes towards glycolytic enzymes, creating an energy crisis that further undermines T‐cell functionality, diminishing activation capacity and cytokine output.^78^ Finally, cell cycle inhibitory proteins are upregulated, thereby driving a proliferation arrest. These systematic alterations at the proteome level collectively lock in the Tex state.

#### 4.4.2 Regulation by PTMs

Post‐translational modification (PTM) proteomics has established the precise, covalent tuning of protein function—typically independent of abundance changes—as a rapid, dynamic regulatory layer and a core mechanism of phenotypic differentiation. This constitutes a core mechanism driving phenotypic differentiation.

##### Phosphorylation

Phosphorylation is the most central and fastest regulatory mechanism. Tex cells exhibit reduced activating phosphorylation levels of key TCR proximal signalling proteins (e.g., ZAP70, LAT and PLCγ1), leading to defective signal transduction. PD‐1 engagement by its ligand PD‐L1 triggers phosphorylation of a tyrosine residue within its intracellular ITSM motif, leading to the recruitment of SHP2 phosphatase. SHP2, in turn, dephosphorylates TCR signalling proteins, directly attenuating TCR signalling^106^ (Figure 5). Conversely, the activating phosphorylation of AKT and mTOR initiates a cascade that culminates in enhanced glycolytic flux, both stimulating the activity of glycolytic enzymes and elevating their expression.

##### Acetylation

Acetylation neutralises the positive charges on histone tails, weakening their interaction with DNA and leading to a transition from a condensed to a relaxed chromatin structure. This open configuration activates gene expression. Deacetylation, catalysed by HDACs, has the opposite effect, promoting chromatin condensation and gene repression. Acetylation thus serves as a critical bridge connecting metabolism, epigenetics and transcription. Beyond histone acetylation, the acetylation of transcription factors (e.g., STAT3 and NF‐κB) directly modulates their DNA binding, stability and activity, thereby altering the transcription levels of genes linked to exhaustion.^86^ Regarding chromatin modifications, the addition of acetyl groups to histone tails (e.g., H3K27ac)—catalysed by HATs and removed by HDACs—is a hallmark of active transcription.^62^

##### Ubiquitination

Ubiquitination critically controls protein stability and turnover.^108^ The E3 ligase CBL‐B, whose activity is often upregulated by tumour cells, drives excessive degradation of TCR components. This attenuates T‐cell signalling, fostering exhaustion and enabling immune escape. In contrast, impaired CBL‐B function in autoimmune settings can lead to hyperactive effector T cells. Given this dichotomous role, pharmacological modulation of E3 ligases like CBL‐B represents a compelling therapeutic avenue. For instance, combining CBL‐B inhibitors with PD‐1 blockade could relieve the multifactorial suppression of T cells in the TME, highlighting a novel strategy for cancer immunotherapy.^109^, ^110^

Collectively, PTMs serve as the final layer of signal execution and integration. They rapidly translate metabolic states (e.g., by using ATP for phosphorylation) and transcriptional commands into precise protein activity, while their own regulation (e.g., by E3 ligases such as CBL‐B) is influenced by the TME, thereby closing the multiomic exhaustion circuit. These PTMs represent the final, rapid‐execution layer of the network. For instance, phosphorylation of AKT in response to microenvironmental signals directly activates mTORC1, reinforcing the metabolic switch to glycolysis detailed in Section 4.3. Conversely, the acetylation of histones and transcription factors establishes a direct link between metabolic enzyme activity (e.g., acetyl‐CoA production) and the epigenetic/transcriptional landscape.

### 4.5 Integrated view: A self‐reinforcing exhaustion circuit

T‐cell exhaustion, from a multiomics standpoint, emerges from a self‐reinforcing circuit that becomes increasingly fixed. The circuit is initiated by chronic antigenic/inflammatory stimulation, which activates drivers like TOX and NFAT^25^ (Section 4.1). These factors are responsible for both instituting the genetic landscape of exhaustion and initiating its epigenetic reprogramming (Section 4.2). These events are coupled to a metabolic switch (Section 4.3) driven by pathways such as the mTORC1 and HIF‐1α pathways.^95^, ^111^ Critically, the new metabolic state feeds back: metabolites such as succinate reinforce the repressive epigenetic scar, which further silences genes needed for oxidative metabolism and effector function. Simultaneously, posttranslational modifications (Section 4.4), such as the phosphorylation of IRs, rapidly attenuate TCR signalling. This renders the cells refractory to stimulation, thereby locking in the exhausted phenotype. Thus, the transition from the Tpex state to the Tex state is a multilayered cascade in which changes at one level (e.g., transcription) induce changes at another (e.g., metabolism), which in turn stabilise and amplify the initial changes, establishing an epigenetically, metabolically and functionally locked state that is resistant to reversal.

### 4.6 From correlation to causality: Functional validation of key targets reveals hubs in exhaustion regulation

In multiomics studies, the molecular landscape of Tpex and Tex cell differentiation has been extensively mapped, revealing a plethora of regulatory factors associated with specific cellular states. However, to establish these molecules as specific drivers of cell fate transition rather than merely correlative markers, rigorous functional validation is indispensable. Targeted gene knockout in cellular or animal models—utilising tools including CRISPR‐Cas9, RNA interference and conditional knockout mice—is fundamental to validating the roles of key regulatory nodes along the Tpex‐to‐Tex differentiation trajectory. This section reviews knockout studies of these critical nodes, aiming to determine their specific functions within the exhaustion regulatory network from a causal perspective. The core findings of these studies are systematically summarised in Table 2.

**TABLE 2:** Functional validation of target genes elucidates the core regulatory network of T‐cell exhaustion.

Target gene (encoded protein)	Functional category	Intervention model	Core phenotype after knockout	Specificity conclusion	Key reference
***Tcf7* (TCF‐1)**	Transcription factor	Conditional KO in antigen‐specific CD8+ T cells during chronic infection	Near‐complete loss of Tpex cells; accelerated terminal exhaustion; severely impaired anti‐tumour persistence	Master regulator for establishing and maintaining the Tpex cell​ reservoir; prevents premature terminal differentiation	[^15^]
***Tox* (TOX)**	Transcription factor	Conditional KO of DNA‐binding domain in T cells during chronic infection	Failure to establish a stable exhausted phenotype; unstable inhibitory receptor expression; increased sensitivity to PD‐1 blockade	Core driver required to initiate and epigenetically 'lock' the exhaustion programm**e**	[^24^]
***Nr4a1/a3* (NR4A1/3)**	Transcription factor	KO in T cells in tumour and chronic infection models	Reduced exhaustion markers in tumour‐infiltrating T cells; enhanced effector function and anti‐tumour capacity	Key synergistic factor for TOX; amplifies and stabilises the transcriptional output of exhaustion	[^19^]
***Prdm1* (BLIMP‐1)**	Transcription factor	Conditional KO in activated CD8+ T cells during chronic infection	Expanded proportion of Tpex cells; blockade of terminal exhaustion differentiation	Negative regulator of Tpex cells; specifically promotes their terminal differentiation	[^81^]
***Dnmt3a* (DNMT3A)**	Epigenetic modifier	Conditional KO in antigen‐specific CD8+ T cells during chronic infection	Enhanced persistence and function of exhausted T cells; improved responsiveness to checkpoint blockade	Epigenetically silences alternative cell fates (e.g., memory/effector programm**e**s) to consolidate the exhausted state	[^112^]
***Ezh2* (EZH2)**	Epigenetic modifier	shRNA‐mediated knockdown in human T cells within an ovarian cancer model.	Upregulation of stemness/memory‐related genes; attenuation of terminal exhaustion features	Imposes repressive H3K27me3 mark to suppress the Tpex cell​ programme, thereby promoting exhaustion	[^113^]
***Ppargc1a* (PGC‐1α)**	Metabolic regulator	Overexpression in virus‐specific CD8+ T cells during early chronic infection	Enhanced mitochondrial and glycolytic function; improved bioenergetic fitness of exhausted T cells	Essential for maintaining metabolic fitness and countering microenvironment‐induced metabolic exhaustion	[^100^]
***Cblb* (CBL‐B)**	E3 ubiquitin ligase	T cell‐specific conditional knockout mice	T cell hyperactivation and autoimmunity tendency; in tumours: enhanced CD8^+^ T cell effector function, persistence and synergy with PD‐1 blockade	E3 ligase that ubiquitinates TCR components as a critical activation checkpoint; its loss breaks negative feedback to enhance responses and counter exhaustion	[^109^]
***Asxl1* (ASXL1)**	Epigenetic regulator	KO CD8+ T cells in chronic infection and tumour models	Sustained Tpex cell​ population; enhanced self‐renewal and effector generation	Epigenetic checkpoint that restrains terminal exhaustion via H2AK119ub modulation, reinforcing T cell stemness and durability	[^112^]
***Etv7* (ETV7)**	Transcriptional regulator	KO CD8+ T cells in tumour and chronic infection models	Failed Tpex cell​ maintenance; reduced transposable element (e.g., VL30) expression; accelerated terminal exhaustion	Promotes Tpex cell​ identity and stemness by driving transposable element expression to inhibit terminal differentiation	[^114^]
***Hmgb2* (HMGB2)**	Transcriptional regulator	KO (P14) CD8+ T cells in chronic infection (adoptive transfer)	Failure to sustain Tpex cell​ pool despite TCF‐1/TOX expression; impaired long‐term survival	Essential for the epigenetic and transcriptional programming underlying Tpex cell​ differentiation and maintenance	[^115^]

*Note*: This table is a synthetic summary. All information presented (including molecular categories, functional impacts and therapeutic implications) was synthesised from the original research papers cited in the ‘Reference’ column for each respective row. These original papers provide detailed evidence, including experimental validation and data analysis. The work of this review did not involve the re‐analysis of raw data from public repositories such as GEO or TCGA.

In summary, systematic functional validation studies have transformed the molecular correlative networks identified by multiomics analyses into a clear map of causal regulation. These experiments have established two core opposing regulatory axes centred on TCF‐1 and TOX, which govern the maintenance of stem‐like plasticity and the lock‐in of terminal exhaustion, respectively. Factors such as NR4A, BATF/IRF4 and BLIMP‐1 provide synergistic or inhibitory inputs at specific stages. Further functional dissection of epigenetic modifiers (e.g., EZH2) and metabolic regulators (e.g., PGC‐1α) has revealed windows of plasticity in the epigenetic and metabolic layers, indicating that the exhausted state is susceptible to multidimensional intervention. These mechanistic insights have directly informed innovative therapeutic strategies. For instance, the rationale for genetic engineering approaches—such as knocking out TOX or overexpressing TCF1 to reprogramme cell fate—is firmly rooted in these functional validations. Looking forwards, employing spatiotemporally specific inducible knockout systems to dynamically resolve gene function will help identify additional upstream initiators of exhaustion and ultimately advance the development of novel therapies capable of precisely reversing T‐cell exhaustion.

## 5 RESEARCH ADVANCES AND THERAPEUTIC PROSPECTS

A suite of powerful new tools has emerged, ranging from high‐resolution multiomics methods like CITE‐seq, scATAC‐seq and scChIP‐seq to sophisticated CRISPR screening and editing platforms, collectively empowering unprecedented investigative depth.^34^, ^91^ These advances have greatly increased our understanding of the differentiation pathway from Tpex cells to Tex cells beyond static lineage relationships towards a dynamic and intricate view of its regulatory network and inherent heterogeneity. This deeper insight has inspired novel therapeutic strategies that are now being actively pursued. These approaches have the dual goal of blocking the Tpex‐to‐exhaustion transition and preserving or restoring effector T‐cell function, representing a new frontier in immunotherapy.

### 5.1 Integrated intervention against T‐cell exhaustion: A multiomics perspective

Building on the multiomics regulatory network delineated in Chapter 4, diverse therapeutic strategies have been devised to interrupt the exhaustion programme. A foundational principle is that modulating the strength of TCR signalling can influence exhaustion, as an optimal signal strength helps maintain Tpex cell stemness, whereas a suboptimal signal strength accelerates terminal differentiation.^116^ In the following sections, these strategies are categorised according to the regulatory layer they primarily target.

#### 5.1.1 Transcription factor intervention strategies

Direct targeting of the core transcriptional circuitry (Section 4.1) constitutes a potent strategy. CRISPR‐based gene editing enables precise manipulation of the expression of key exhaustion drivers, for example, allowing the knockdown of TOX/TOX2 or NR4A to suppress the exhaustion programme or the overexpression of c‐Jun and TCF1 to enhance effector function and stem‐like properties, thereby reprogramming T‐cell fate.^19^, ^43^, ^80^, ^117^ Other factors, such as BATF and BACH2, are also emerging as critical nodes for intervention.

#### 5.1.2 Epigenetic reprogramming

The Tex state, once considered irreversible, is now recognised as being partially reversible, challenging the traditional dogma and validating the therapeutic concept of reprogramming the epigenetic scar (Section 4.2). Exposure to cytokines like IL‐2 or specific pharmacological agents can reprogramme a fraction of Tex cells, enabling them to regain a Tpex‐like state and partial function.^118^ Agents targeting epigenetic modifiers—for instance, the HDAC inhibitor chidamide and the DNMT inhibitor decitabine—can partially dismantle the locked epigenetic state, restore effector capacity and lower the levels of exhaustion markers, including PD‐1.^41^ The inhibition of other targets, such as ENOA1, has also shown promise in reversing the exhaustion signature.^25^ A core principle is that the early establishment of exhaustion demands equally early therapeutic interception.

In support of the druggability of epigenetic nodes, genetic studies have identified key regulators whose manipulation profoundly affects exhaustion. In patients, somatic mutations in genes such as *Asxl1* are associated with long‐term survival following immunotherapy.^112^ Preclinical evidence indicates that ablating key epigenetic regulators Asxl1—bolsters T‐cell durability in chronic infections and sustains their sensitivity to checkpoint inhibition. This positions these regulators as compelling candidates for genetic modification, providing a rationale for their use in advanced cell‐based immunotherapies.^112^

Beyond the established regulators, novel targets continue to be discovered. For example, recent studies highlight HMGB2 as a driver of the Tpex‐specific transcriptional programme, mediated through its role in shaping chromatin accessibility.^115^ This makes it a novel and promising target for immunotherapy.

#### 5.1.3 Metabolic and microenvironmental reprogramming

Current research indicates that reprogramming mitochondrial metabolism can counteract T‐cell exhaustion. Strategies to reverse exhaustion at the metabolic level include improving mitochondrial function (e.g., via PGC1α agonists), providing alternative fuels (e.g., acetate and fatty acids) and alleviating endoplasmic reticulum stress (e.g., via IRE1/PERK inhibitors).^119^, ^120^, ^121^ Clearing metabolites from the TME can slow the exhaustion process. A synergistic strategy combines ICB with metabolic interventions, as ICB itself can partially correct metabolic defects.^122^ Supportive approaches to reprogramme T‐cell metabolism include: employing antioxidants like N‐acetylcysteine (NAC) to neutralise ROS^123^; activating AMPK via metformin to revive oxidative metabolism; and supplementing with NAD+ precursors to enhance mitochondrial fitness.^26^, ^124^ Engineering T cells to overexpress mitochondrial fusion proteins such as MFN2 or to express glycolytic inhibitors can help maintain a healthier metabolic state in vivo, thereby delaying exhaustion.^31^ Mitochondria‐targeted antioxidants like MitoQ can enhance mitochondrial fitness, thereby boosting the anti‐tumour potency of Tex cells.^125^ Inhibiting key glycolytic enzymes such as PKM2 can also decelerate the progression of exhaustion.^125^ Thus, modulating glycolysis via metabolic interventions represents a viable strategy with the dual objectives of postponing exhaustion and enhancing both the potency and durability of T‐cell responses.

Tumour cells and some immune cells produce large amounts of lactate, resulting in an acidic TME. Studies have shown that MCT1‐mediated lactate uptake exacerbates T‐cell dysfunction.^47^ This mechanistic understanding opens a new front for enhancing immunotherapy, namely the targeting of tumour metabolism. One approach is the development of small‐molecule MCT inhibitors to block lactate uptake. This concept is strengthened by separate findings that reprogramming mitochondrial metabolism is effective against exhaustion in CAR‐T cells.^126^

#### 5.1.4 Proteostatic and PTM‐targeted interventions

A unique proteotoxic stress response has emerged as a defining and mechanistically active feature of exhausted T cells. This discovery highlights a novel therapeutic concept: restoring T‐cell function by intervening in cellular protein quality control pathways. For instance, targeting the chaperone system or modulating autophagy may attenuate this stress response, thereby potentiating the antitumour immune capacity of T cells and opening a new strategic landscape.^127^

Moving beyond traditional antibody blockade, an emerging therapeutic paradigm exploits the posttranslational modification of immune checkpoint proteins to induce their degradation—a strategy that actively depletes the targets rather than merely blocking their function. Research in this area prominently focuses on the modification landscapes of PD‐1 and PD‐L1. Concurrently, the palmitoylation of TIM‐3 has been identified as a positive regulator of T‐cell exhaustion.^128^ Proof of this principle is demonstrated by a peptide inhibitor that disrupts TIM‐3 palmitoylation, which promotes the receptor's degradation and consequently potentiates the anti‐tumour immune response. Together, these advances establish the targeted regulation of PTMs as a novel and promising axis for therapeutic intervention against T‐cell exhaustion.

#### 5.1.5 Immune checkpoint blockade and rational combination therapies

First‐generation ICB comprises monotherapies using agents directed against PD‐L1 or PD‐1. Evidence shows that blockade of this axis can reactivate the Tpex population and stimulate their proliferation. Consequently, the frequency of Tpex cells can predict patient responsiveness to ICB therapy.^3^ However, monotherapies have a limited response rate, and they often fail to reverse the deep‐seated epigenetic exhaustion state. Studies have shown that CD28 co‐stimulation critically governs the functional fate of T cells.^129^ The functional competence of T cells following PD‐1 blockade is not fully restored without sufficient co‐stimulation. Thus, strategies that combine checkpoint inhibition with co‐stimulatory agonists are necessary to achieve optimal therapeutic outcomes.

Current clinical strategies frequently combine ICB with various other modalities. To enhance the benefit of ICB, therapeutic strategies have evolved towards the concurrent blockade of multiple pathways, such as combined targeting of the PD‐1 axis and complementary inhibitory pathways, thereby achieving more complete T‐cell restoration.^51^ Combinations of ICB agents with agonists of co‐stimulatory receptors (e.g., anti‐4‐1BB and anti‐OX40 antibodies) provide potent activating signals.^46^ Combination approaches further encompass strategies that modulate soluble mediators. These strategies are aimed at distinct functional goals: enhancing stimulatory signals, counteracting immunosuppressive factors and interrupting adenosine‐mediated suppression.^18^, ^120^, ^130^ The efficacy of immunotherapy can be further amplified by integrating it with conventional modalities like radiotherapy or chemotherapy. A particularly synergistic paradigm involves combining checkpoint blockade with adoptive cell therapies. In this approach, engineered T cells—while potent against hematologic cancers—often face functional suppression within solid TMEs. Co‐administration of PD‐1/PD‐L1 inhibitors can mitigate this suppression, thereby enhancing the longevity and functional persistence of these therapeutic cells. Preclinical evidence supports this strategy: augmenting PD‐1 blockade in models with T cells engineered to overexpress c‐Jun yields significantly stronger anti‐tumour activity.^117^

Notably, inhibitory immune cells such as Tregs are key drivers of exhaustion and can impair responses to ICB, indicating the importance of considering combination strategies that target Tregs.

#### 5.1.6 Next‐generation engineered cell therapies: Engineering exhaustion resistance

A central objective in designing next‐generation cell therapies is to genetically engineer T cells to maintain a Tpex‐like stem cell state or prevent their differentiation into Tex cells. Strategies that target key transcription and epigenetic factors inducing exhaustion—that is, gene knockout, protein degraders and inhibitory proteins—can empower CAR‐T cells with greater longevity and enhanced therapeutic potency.

The most straightforward optimisation strategy involves direct editing of the core transcriptional circuits that govern exhaustion in therapeutic T cells. This includes targeting established drivers of exhaustion, such as TOX and NR4A, as well as recently defined key determinants like ETV7, which acts to lock in the terminal exhaustion programme.^19^, ^114^ Conversely, enforcing the expression of stemness‐associated factors (e.g., TCF‐1) promotes a progenitor‐like state, thereby enhancing persistence.^15^ These modifications directly intercept the molecular pathways leading to functional decline.

Beyond genomic editing, the exhausted phenotype can be counteracted by pharmacological remodelling the epigenetic landscape. A clinical approach combines adoptive cell therapy with epigenetic modulators—for instance, administering a DNMTs inhibitor like decitabine or HDAC inhibitors around the time of infusion.^131^ This can reshape both the immunosuppressive tumour niche and the therapeutic T cells’ own epigenome, creating conditions less conducive to exhaustion.

A third strategy seeks to broadly enhance intrinsic cellular fitness, rendering engineered T cells more resistant to triggers of dysfunction. Functional genomics provides clues: an in vivo CRISPR screen revealed that CDKN1B deletion markedly improves the long‐term expansion and functional capacity of these cells, indicating its role in promoting a an exhaustion‐resistant state. ^132^

In summary, the various omics layers are interconnected in regulating the process of differentiation from Tpex cells to Tex cells, resulting in the formation of a complex regulatory network. In clinical therapy, combining interventions targeting different aspects of this network can yield greater therapeutic efficacy.

### 5.2 Translational challenges for therapeutic strategies

Currently, various strategies aimed at reversing T‐cell exhaustion through distinct mechanisms are continuously being developed. While these strategies demonstrate unique potential, they also face significant challenges in clinical translation. Epigenetic reprogramming may offer durable reversal of the exhaustion programme, but it is hampered by key obstacles such as low delivery efficiency and poor targeting specificity, with limited intervention efficacy often observed in patients with advanced‐stage disease.^41^ Metabolic drugs are highly accessible, yet tumour metabolic heterogeneity can readily lead to drug resistance, and the narrow therapeutic window of these drugs poses a substantial risk of systemic toxicity.^26^ Although transcription factor‐targeted interventions are capable of precisely targeting key regulatory nodes, achieving accurate modulation with these drugs is extremely difficult because of the complexity and redundancy of regulatory networks, as well as the pleiotropic functions of pivotal factors. Proteome‐based approaches constitute a novel perspective, but the targets of these approaches generally lack T‐cell specificity, and major bottlenecks in drug delivery and stability remain.^128^ Combination immunotherapies targeting immune checkpoints can increase response rates, but the use of these combinations is accompanied by increased toxicity, high costs and a heavy reliance on precise patient stratification.^3^ Therefore, future breakthroughs likely depend on rationally designed multidimensional combination strategies, although the design and validation of such approaches are still in their early stages.

At the technical translation level, precision interventions face bottlenecks. For both gene‐editing tools and small‐molecule drugs, issues such as low targeted delivery efficiency, high off‐target risks and potential safety concerns persist. The use of engineered cell therapies is limited in solid tumours because of insufficient persistence, the complexity of genetic modifications and adverse effects, limiting their broad application.^26^

At the clinical implementation level, patient heterogeneity is a key obstacle. There is currently a lack of dynamic, integrated biomarkers capable of reliably predicting therapeutic responses, resulting in variable efficacy and high failure rates in clinical trials. Furthermore, patients with varying degrees of T‐cell exhaustion exhibit differential therapeutic sensitivity, a nuance not adequately addressed by current treatment paradigms.^16^ Many combination therapies are empirically rather than mechanistically designed, often leading to increased toxicity without proportional gains in efficacy.

Notably, a significant challenge lies in bridging the persistent gap between preclinical knowledge and human application. Our mechanistic knowledge of T‐cell exhaustion, still predominantly shaped by murine studies, must be effectively applied to the development of clinically relevant therapies.

## 6 CONCLUDING SUMMARY AND FUTURE PERSPECTIVES

The differentiation process that begins with Tpex cells and culminates in terminal exhaustion is not a linear event confined to a single regulatory layer but rather a self‐reinforcing feedback loop orchestrated by a multitiered network involving transcriptional, epigenetic, metabolic and posttranslational modifications. This process begins with transcriptional reprogramming, where the antagonism between the TCF‐1‐mediated stemness programme and the TOX/NR4A/BATF‐driven exhaustion programme establishes the initial directive for cell fate commitment. This transcriptional instruction is further executed and amplified through metabolic reprogramming: a metabolic rewiring that prioritises aerobic glycolysis over OXPHOS and FAO. This serves dual roles: provisioning the necessary energy and biosynthetic building blocks and concurrently generating signalling metabolites such as α‐KG, which directly modulate the activity of epigenetic modification enzymes. Consequently, a stabilised epigenetic landscape is gradually established, thereby locking in the exhaustion transcriptional programme dominated by factors such as TOX. Simultaneously, fine‐tuning of the expression of key proteins through posttranslational modifications, including phosphorylation and acetylation, further modulates and consolidates this state. Studies employing single‐cell multiomics technologies have revealed that it is precisely these continuous and bidirectional interactions across layers—for instance, metabolites shape the epigenetic landscape, which in turn feeds back to regulate transcription and metabolism—that collectively form a selfreinforcing exhaustion network, irreversibly driving cells from a plastic state towards functional lock‐in.

Translating this mechanistic map into durable therapies remains a central challenge. Current strategies, which often target single regulatory layers, are limited by the inherent complexity of the system and patient heterogeneity. Future progress hinges on a fundamental conceptual transition: moving beyond static observation towards a dynamic, understanding focused on three core aspects. First is the move from correlation to causality: We must move beyond descriptive maps to establish causal mechanisms within the multilayered network. This requires technologies for the simultaneous perturbation of transcriptional, epigenetic and metabolic nodes coupled with real‐time, multidimensional readouts, enabling the construction of predictive, dynamic models of T‐cell fate decisions. Second is the decoding of the microenvironmental instructor: The tissue‐specific microenvironment is the primary instructor of exhaustion. A pivotal objective is to decipher, with spatial precision, how the microenvironment—through stromal cells, metabolites and biophysical forces—actively moulds the T‐cell state. This insight is fundamental for developing targeted, context‐specific therapies. Last is the definition of the thresholds for reversal: Given the stability of the exhausted state, defining the critical thresholds for reversibility is paramount. This involves identifying mechanistic biomarkers of plasticity and employing computational models to predict optimal, sequential intervention sequences, thereby enabling the design of rational, precise combination therapies.

Addressing these priorities requires a co‐ordinated methodological evolution integrated with clinical translation. This path includes leveraging dynamic multiomics profiling and advanced in *vivo* imaging approaches to generate the necessary high‐resolution longitudinal and spatial data, developing more physiologically relevant human model systems (e.g., advanced humanised mice and organoids) to bridge the gap between preclinical discovery and human immunology, and pioneering biomarker‐driven adaptive clinical trial designs that can dynamically match evolving therapeutic strategies to patient subsets defined by multidimensional biomarkers (e.g., TCF‐1^+^ frequency, *ASXL1* status and transposable element expression).

The collective goal of this endeavour is a paradigm shift from broad immunosuppressive blockade to precision immune reprogramming. The future lies in learning to dynamically reprogramme the regulatory network in exhausted cells in *situ*—not merely to transiently relieve inhibition but also to actively steer T‐cell fate towards a durable, functional and protective state. The actualisation of this vision offers a novel and powerful path towards successfully treating chronic infections and cancer.

## AUTHOR CONTRIBUTIONS

**Tong Zhu**: Writing—original draft. **Xiaoyu Teng**: Validation. **Qinlian Jiao**: Visualisation. **Yidan Ren**: Validation. **Yunshan Wang**: Supervision. **Maoxiao Feng**: Writing—review and editing.

## ETHICS STATEMENT

This article is a review of previously published literature and does not contain any new studies with human participants or animals performed by any of the authors. Therefore, no separate ethical approval was required for this work.

## CONFLICT OF INTEREST STATEMENT

The authors declare no conflicts of interest.

## Data Availability

Data sharing is not applicable to this article as no datasets were generated or analysed during the current study.
